# Exploring the relationships among safety leadership, safety climate, psychological contract of safety, risk perception, safety compliance, and safety outcomes

**DOI:** 10.3389/fpubh.2023.1235214

**Published:** 2023-10-23

**Authors:** Leila Omidi, Hossein Karimi, Colin Pilbeam, Saeid Mousavi, Gholamreza Moradi

**Affiliations:** ^1^Department of Occupational Health Engineering, School of Public Health, Tehran University of Medical Sciences, Tehran, Iran; ^2^Department of Health, Safety, and Environment, School of Health, Tabriz University of Medical Sciences, Tabriz, Iran; ^3^Cranfield Safety and Accident Investigation Centre, Cranfield University, Cranfield, United Kingdom; ^4^Department of Statistics and Epidemiology, School of Health, Tabriz University of Medical Sciences, Tabriz, Iran; ^5^Department of Occupational Health Engineering, School of Health, Tabriz University of Medical Sciences, Tabriz, Iran

**Keywords:** safety leadership, safety climate, psychological contract of safety, deep compliance, surface compliance

## Abstract

**Background:**

Recently, two types of safety compliance behaviors including deep compliance and surface compliance were differentiated. The current study aimed to investigate the relationships among safety leadership, safety climate, psychological contract of safety (PCS), risk perception, and deep compliance and surface compliance behavior of workers. In addition, the effects of both deep and surface compliance on safety outcomes were considered.

**Methods:**

Workers’ perceptions in terms of safety leadership, safety climate, PCS, risk perception, deep compliance, and surface compliance were measured by appropriate questionnaires. Three questions were asked to measure undesired safety outcomes. Structural equation modeling and correlation analysis were applied to examine the research model and relationships among variables.

**Results and discussion:**

The results of the current study showed that deep compliance was positively predicted by safety leadership, safety climate, and PCS and negatively predicted by risk perception. Surface compliance was positively predicted by safety leadership and safety climate and negatively predicted by risk perception. Surface compliance is not significantly predicted by PCS. With regard to the adverse safety outcomes, the results showed that both deep and surface compliance were negatively associated with adverse safety outcomes, however, deep compliance had a stronger negative correlation with adverse safety outcomes than surface compliance.

## 1. Introduction

Steel manufacturing is a risky workplace and safety is an important concern in steel-manufacturing industries. Workers in the steel-manufacturing context face many types of risks which are related to the nature of the job and working environment. Heavy work tasks, hot and noisy environments, crushing injuries, and burns are some of the work-related stressors experienced by metalworkers (1).

Workers’ unsafe behavior appears to be a key factor in work-related accidents in high-risk industries. Workplace accidents impose a large direct cost and indirect damage such as injuries and psychological costs for employees (2). In addition, a lack of compliance with safety rules and procedures is identified as a central contributory factor to work-related accidents in accident investigations (3).

Previous studies have shown that safety leadership, perceived safety obligations, perceived safety climate level, and risk perception impact workers’ safety behavior (2, 4, 5).

Safety leadership plays a key role in supporting safety in workplace settings. Leader-Member Exchange and transformational leadership can affect workers’ safety participation behaviors and their voluntary participation in safety-related activities (6). Clarke (7) suggested that safety leadership is important in ensuring compliance with safety rules and regulations. Also, leader behaviors affect subordinates’ safety behaviors. Safety leadership has an important influence on shaping workers’ perceptions regarding the importance of safety in the workplace. Two aspects of safety leadership including transactional leadership and transformational leadership have been proven to be effective in workers’ safety compliance. Transactional leaders clarify expectations, roles, and task requirements and recognize the actions subordinates must take to achieve outcomes to fulfill leader expectations and achieve outcomes (7, 8). This type of leadership is important in ensuring compliance with safety regulations and rules. Transformational leaders as intellectually stimulating leaders help subordinates develop new ways of problem solving. Transformational leadership is positively associated with employees’ safety participation and their perceived safety climate (7).

Safety climate refers to the employees’ shared perceptions regarding an organization’s policies, practices, and procedures in relation to safety issues that demonstrate the priority of safety (9–12). Previous studies have suggested the positive relationship between safety leadership and safety climate (7), safety climate and psychological contract of safety (5), and safety climate and safety compliance (13). Safety climate has a significant negative association with risk perception (14).

Psychological contract theory is developed based on social exchange theory and considers a perceived exchange relationship between an employer/supervisor and an employee. A psychological contract of safety (PCS) suggested the beliefs of employees about reciprocal safety obligations in the workplace. Empirical evidence revealed that perceived employer/ supervisor safety obligations can positively influence employees’ safety behavior and PCS can be a key factor in describing how employees attach meaning to employer/ supervisor behavior in their workplace. Perceived employer/supervisor breach or fulfillment of the psychological contract of safety has also been examined in the domain of leader-member exchange studies (4, 5, 15).

The extent of employees’ exposure to danger while working is defined as workplace risks and the employees’ subjective judgment of the risk is referred to risk perception (16, 17). Risk perception is associated with the employees’ assessment regarding the probability and severity of the undesired effects occurring (such as accidents and injuries) (18). The link between risk perception and safety behavior is confirmed in the previous finding concerning the influence of risk perception on safety behavior (2). In recent years, the importance of leaders in enhancing workers’ safety behaviors was mentioned by safety researchers. Safety leadership can also influence the level of workers’ perceived risk. There is a negative link between risk perception and safety leadership (18).

Safety behaviors are related to some activities ensuring that the workplace is free from harm or physical threat. Based on job performance theory and regarding the task and contextual work performance, two components of safety behavior are distinguished in the previous studies that include safety compliance and safety participation. Safety compliance is associated with some behaviors such as doing work in a safe manner and adhering to safety rules and procedures. Safety participation involves behaviors like workers’ voluntary participation in safety activities and putting efforts into enhancing safety in the workplace settings (19). Hu et al. (20) found that perceived organizational support for safety and perceived supervisor support for safety can impacts compliance with safety procedures and perceived ease of use and perceived usefulness are some antecedents of safety compliance. The traditional perspective in safety science supposes that compliance behavior is a unidimensional construct. Recently, Hu et al. (21) distinguished two forms of compliance behaviors including surface compliance and deep compliance based on the concepts of surface and deep acting with regard to the emotional labor literature. Workers engage in surface compliance behaviors with the aim of meeting organizational requirements and direct their effort and attention toward illustrating compliance. Workers engage in deep compliance behaviors with the aims of maintaining workplace safety and investing the effort needed for enacting risk management strategies expected to achieve desired organizational safety outcomes. It is found that surface compliance was negatively associated with management commitment to safety, positively associated with punishment climate, and also positively related to undesired safety outcomes (accident, injury, and near miss). Safety outcomes is defined as an event or results of employees’ safety performance and commonly refers to accidents, injuries, and near misses. It was found that deep compliance was negatively associated with undesired safety outcomes. Employees exhibiting deep compliance invest sufficient effort into achieving desired safety goals (21, 22).

### 1.1. Objectives and hypotheses

**Figure 1:**
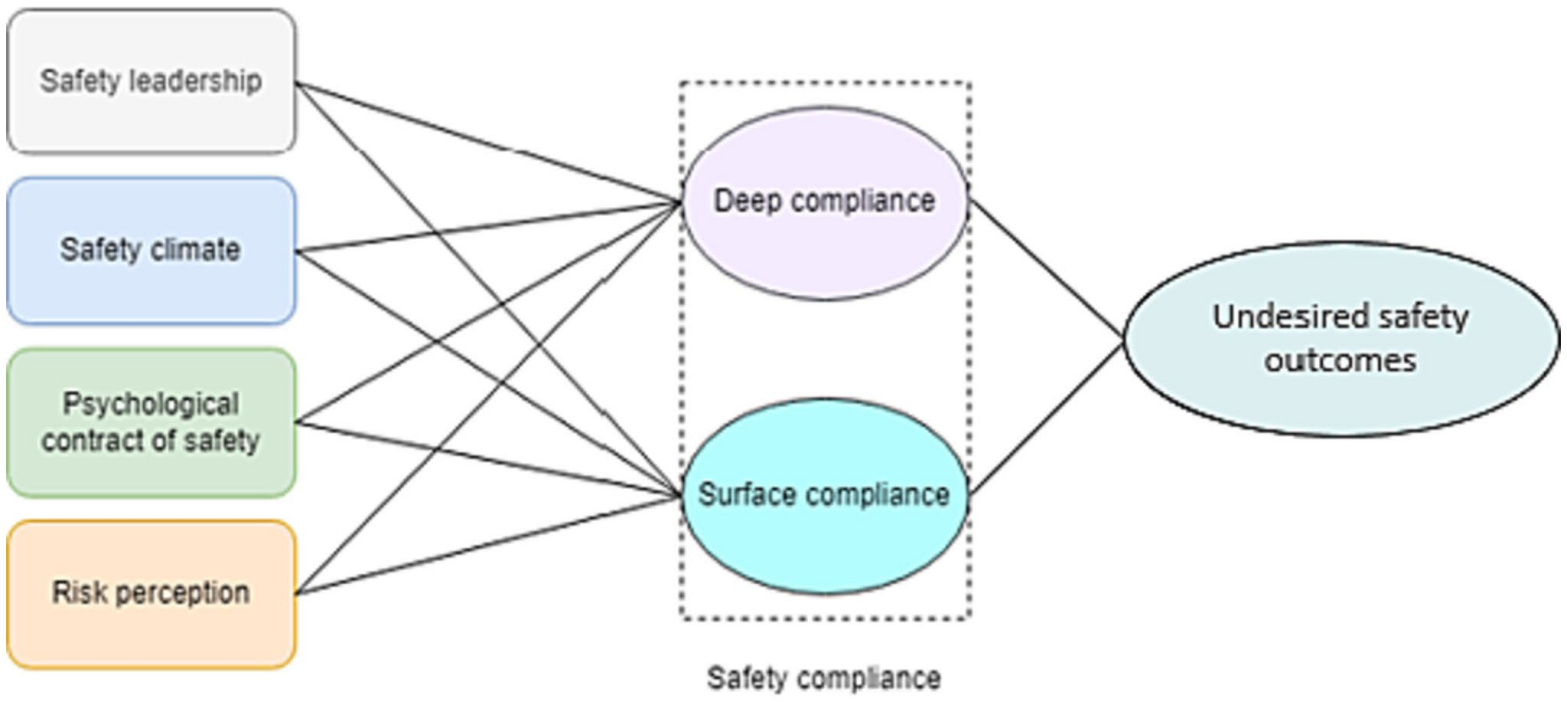
Research model.

The current study aimed to investigate the relationships among safety leadership, safety climate, psychological contract of safety, risk perception, and two types of safety compliance behavior including deep compliance and surface compliance. In addition, the effects of both deep and surface compliance on safety outcomes (accidents, injuries, and near misses) were taken into account. Figure 1 presents the hypothesized model of the current study. We hypothesize that:

*H1a*: Safety leadership significantly influences deep compliance.

*H1b*: Safety climate significantly influences deep compliance.

*H1c*: Psychological contract of safety significantly influences deep compliance.

*H1d*: Risk perception is negatively related to deep compliance.

*H2a*: Safety leadership significantly influences surface compliance.

*H2b*: Safety climate significantly influences surface compliance.

*H2c*: Psychological contract of safety significantly influences surface compliance.

*H2d*: Risk perception is negatively related to surface compliance.

*H3a*: Deep compliance is negatively related to undesired (adverse) safety outcomes.

*H3b*: Surface compliance is negatively related to undesired safety outcomes.

## 2. Method

### 2.1. Participants

Cross-sectional survey data were gathered from 250 workers of a steel-manufacturing company in Iran. A total of 205 workers answered the survey. All of them were male. Among study workers, about 61% were in the age range of 41–50 years, 24% were in the age range of 31–40 years, and 10% were in the age range of 51–60 years. Approximately, 56% of them had 16–20 years of work experience and 28% had more than 20 years of work experience. About 44% of workers had a Bachelor of Science degree and 6% had a Master of Science degree.

### 2.2. Measures

Items were measured using a five-point Likert scale (1 = strongly disagree and 5 = strongly agree).

Workers’ perceptions in terms of safety leadership were measured using 10 items adapted from Avolio et al. (23) and Fernández-Muñiz et al. (24). Example items are “The managers/supervisors recognize workers’ achievement to safety goals,” and “The managers/supervisors clarify rewards for compliance with safety procedures.” The Cronbach alpha for this sample was 0.74.

Safety climate was assessed by three items developed by Neal and Griffin (19). An example item is “Safety is given a high priority by management.” The Cronbach alpha for this sample was 0.81.

PCS was assessed by 12 items adapted from Newaz et al. (5). Example items are “My manager/supervisor meets their obligation to listen to employee safety concerns” and “My manager/supervisor meets their obligation to set a good example for safety behavior.” The Cronbach alpha for this sample was 0.82.

Risk perception was measured by 3 items adopted from Oah et al. (18). An example item is “In my workplace, the chances of being involved in an accident are quite large.” The Cronbach alpha for this sample was 0.76.

Deep compliance was measured by 5 items adopted from Hu et al. (21). Example items are “I tried to be safe by carrying out each step of the procedure with my full attention” and “I tried to work safely by foreseeing how my actions could impact safety.”

Surface safety compliance behavior was assessed by 3 items adapted from Neal and Griffin (19). An example item is “I use the correct safety procedures for carrying out my job.” The Cronbach alpha for this sample was 0.80.

For measuring undesired safety outcomes, participants were asked to answer the questions about the history of accidents, injuries, and near misses over the past 12 months (21). Three questions were asked. An example item is “How many work-related accidents have you been involved in over the past 12 months?”

### 2.3. Data analysis

Statistical package for social science (SPSS 20) and analysis of moment structure software (AMOS 24) were employed for data analysis. Structural equation modeling (SEM) was applied for examining the research model and relationships among the observed and latent variables. To test the overall fit of the model, fit indexes such as *χ*^2^ ratio (< 3), CFI (comparative fit index) (> 0.90), and RMSEA (root mean square error of approximation) (< 0.08) were employed. To examine the correlations among the variables, the Spearman correlation coefficient was applied.

## 3. Results

The results of correlation coefficient analysis demonstrate significant positive correlations between employees’ deep compliance and safety leadership (*r* = 0.16, *p* < 0.01), safety climate (*r* = 0.27, *p* < 0.01), and PCS (*r* = 0.17, p < 0.01). Also, significant negative correlation was observed between deep compliance and risk perception (*r* = − 0.19, *p* < 0.01) and deep compliance and undesired safety outcome (accidents, injuries, and near misses) (*r* = − 0.15, *p* < 0.01). Significant positive correlations were observed between surface compliance and safety leadership (*r* = 0.13, *p* < 0.01) and between surface compliance and safety climate (r = 0.23, *p* < 0.01). Furthermore, negative correlations were observed between surface compliance and risk perception (*r* = − 0.15, *p* < 0.01) and between surface compliance and undesired safety outcomes (*r* = − 0.10, *p* < 0.05). Moreover, there was a significant positive relationship between employees’ deep compliance and surface compliance (*r* = 0.37, *p* < 0.01) (Table 1).

**Table 1:** Correlations among variables.

Variable	1	2	3	4	5	6	7	8	9	10
1. Age	–									
2. Work experience	0.47^**^	–								
3. Education level	0.08	0.21^**^	–							
4. Safety leadership	0.09	0.17^**^	0.10	–						
5. Safety climate	0.03	0.07	0.03	0.57^**^	–					
6. PCS	0.08	0.12	0.15^**^	0.66^**^	0.58^**^	–				
7. Risk perception	0.23^**^	0.17^*^	0.11	−0.12^**^	−0.18^**^	−0.16^**^	–			
8. Deep compliance	0.05	0.07	0.10	0.16^**^	0.27^**^	0.17^**^	−0.19^**^	–		
9. Surface compliance	0.05	0.11	−0.15^*^	0.13^**^	0.23^**^	0.10	−0.15^**^	0.37^**^	–	
10. Safety outcomes	−0.10	−0.13	−0.02	−0.12^*^	−0.14^*^	−0.11	−0.25^**^	−0.15^*^	−0.10	–

^**^Correlation is significant at the 0.01 level; ^*^Correlation is significant at the 0.05 level; and PCS is psychological contract of safety.

**Figure 2:**
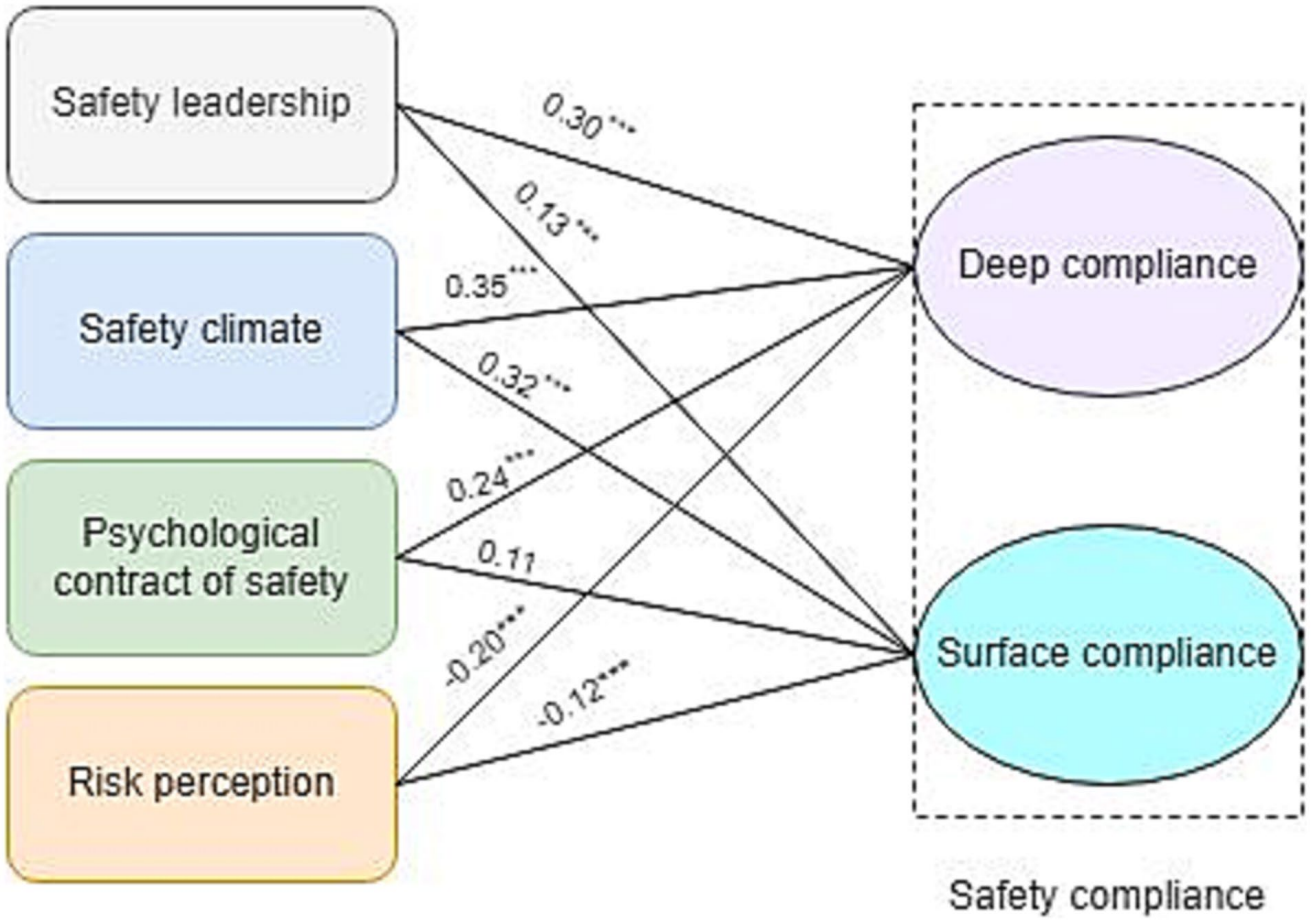
Results of the proposed model. ^***^Correlation is significant at the 0.001 level.

**Figure 3:**
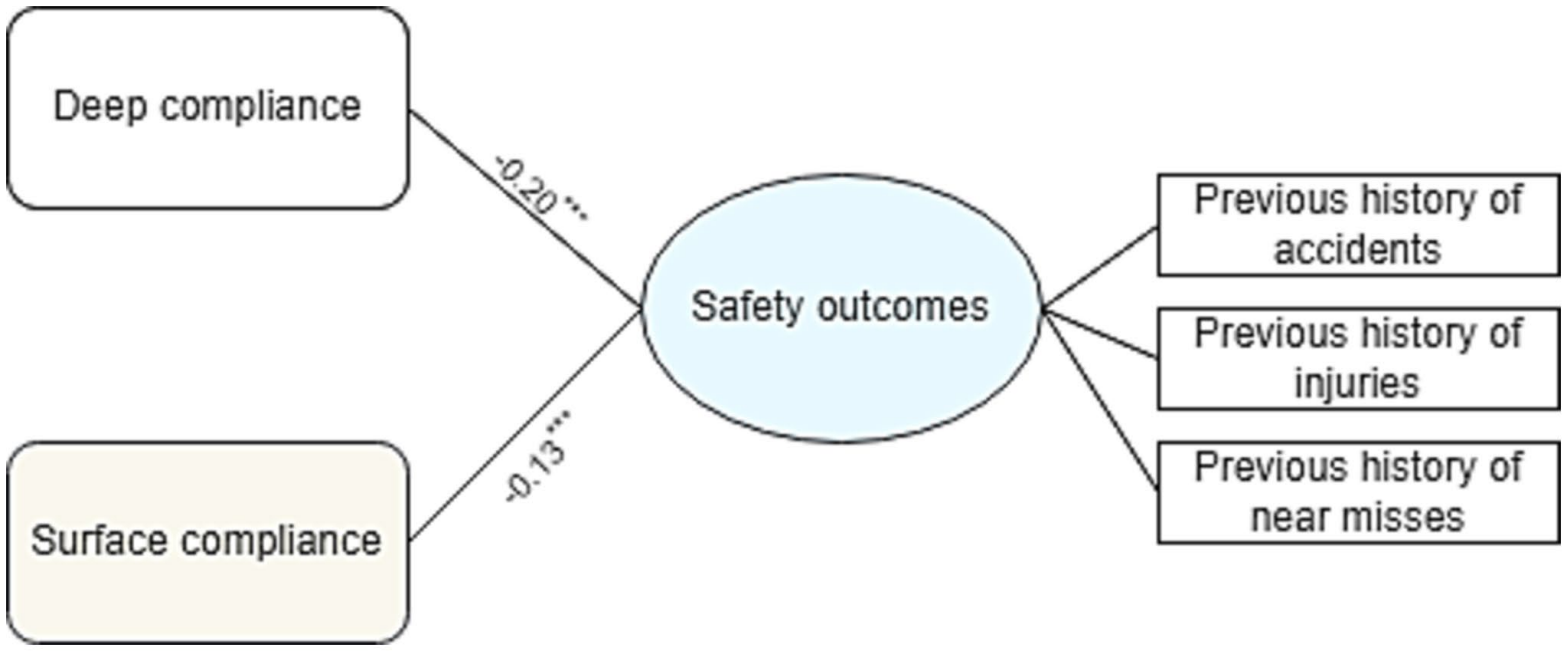
Effects of two forms of safety compliance behavior on undesired safety outcomes. ^***^Correlation is significant at the 0.001 level.

The results of SEM analysis for testing the proposed model suggested that the proposed model has a very good fit to the data in the current study (Chi-square/df = 1.23, CFI = 0.96, and RMSEA = 0.04). Figure 2 illustrates the significance of safety leadership (*β* = 0.30, *p* < 00.1), safety climate (*β* = 0.35, *p* < 0.001), and PCS (*β* = 0.24, *p* < 0.001) in predicting employees’ deep compliance. Both deep compliance (*β* = − 0.20, *p* < 0.001) and surface compliance (*β* = − 0.12, *p* < 0.001) were negatively related to risk perception (Figure 2). Based on the results, hypotheses H1a to H1d, H2a, H2b, H2d, H3a, and H3b were supported by these findings in that safety leadership, safety climate, and psychological contract of safety have significant positive influences on deep compliance, safety leadership and safety climate have significant positive influences on surface compliance, and risk perception has a negative significant influence on both deep compliance and surface compliance. Among all hypotheses, H2c was not supported. That is, the psychological contract of safety had no significant impact on surface compliance. In addition, both deep (*β* = − 0.20, *p* < 0.001), and surface compliance (*β* = − 0.13, *p* < 0.001), were negatively associated with undesired safety outcomes (Figure 3), however, deep compliance had a stronger negative correlation with adverse safety outcomes than surface compliance.

## 4. Discussion

The previous study differentiated two different forms of safety compliance behavior including deep and surface compliance regarding complying with safety procedures. Moreover, it is suggested that surface compliance increases the risks of adverse safety outcomes and deep compliance reduces the risk of undesired safety events (21). The current study was conducted to assess the relationships among safety leadership, safety climate, psychological contract of safety, risk perception, and two forms of safety compliance behavior including deep compliance and surface compliance. In addition, the effects of both deep and surface compliance on safety outcomes were considered.

The results of this study indicated that both forms of safety compliance behavior are positively predicted by safety leadership and safety climate. Clarke (13) showed that safety leadership has significant effects on safety compliance and a perceived safety climate mediates the relationship between safety leadership and safety compliance. Furthermore, Pilbeam et al. (25) indicated that safety leadership practices can affect organizational safety compliance. Leaders’ safety behavior is an influential factor in shaping worker safety performance (safety compliance and safety participation) and may ensure compliance with safety procedures (6).

Hu et al. (21) reported a negative association between surface compliance and management commitments to safety (as a dimension of safety climate). The findings of the current study differ from their findings. In the current study, safety climate is significantly associated with both deep compliance and surface compliance. A possible explanation for this might be the impact of context. The study of Hu et al. (21) was done in different organizations and various occupational backgrounds while the current one was conducted in a single industry having a defined framework of safety procedures and rules for occupational groups in the company and workers are familiar with company safety policy and workplace practices.

It is suggested that the intention of workers to engage in deep compliance is associated with maintaining workplace safety and reducing the efforts needed for effective risk management strategies to achieve desired organizational safety outcomes and the intention of workers to engage in surface compliance is related to meet the requirements of the organization and direct efforts toward demonstrating compliance (21). This study revealed that both deep and surface compliance are important in complying with safety procedures. Although deep compliance tended to correlate most strongly with safety leadership and safety climate, both deep and surface compliance required attention for accomplishing desired safety outcomes, as demonstrated by Yeo and Frederiks (26).

Deep compliance is positively predicted by PCS but this is not so for surface compliance. Deep compliance refers to mindful awareness and careful application of safety procedures and contains a four-stage psychological process encompassing health and risk awareness, perceived utility, behavioral adaption, and integration. Surface compliance indicates compliance with minimal effort (27). As mentioned by Walker (28), one important factor in employee safety obligations is compliance with safety. Following safety rules, complying with safety procedures considering risks and hazards, and proper use of work equipment are essential for employee safety obligations. In addition, setting a good example for safe behavior and providing details about safety procedures are some influential items in PCS (5).

Deep compliance is related to changes in the awareness and perceptions of workers (27). PCS can influence the perception of employees and can predict workers’ safety perception and safety behavior (5). Given that deep compliance affects the awareness and perceptions of employees and consistent with previous findings (27), in the current study, a significant positive relationship was reported between deep compliance and PCS. Conversely, no significant relationship was found for surface compliance and PCS.

Risk perception had significant negative relationships with both deep compliance and surface compliance. Results of previous studies showed negative correlation coefficients among risk perception, safety compliance, and safety participation. Xia et al. (2) suggested a negative correlation between safety compliance and risk perception (*r* = − 0.234^**^, *p* < 0.01). It was found that risk perception as a job hindrance can affect construction workers’ safety behavior and can result in reduced safety motivation and subsequently reduced levels of safety compliance and safety participation behaviors. In addition, as demonstrated by Oah et al. (18), safety climate of organizations and safety leadership were negatively related to risk perception.

In this study, both deep and surface compliance were negatively correlated with undesired safety outcomes. Some parts of the current results differ from those found by Hu et al. (21). Although consistent results were obtained for deep compliance (negative relationship between deep compliance and undesired safety outcomes in both studies), the results were different regarding surface compliance.

In the current study, surface compliance was negatively related to undesired safety outcomes. In the study conducted by Hu et al. (21), positive associations were reported between surface compliance and undesired safety outcomes (*r* = 0.16^*^, *p* < 0.05). Safety compliance is considered an observable safety behavior that can have positive impacts on organizational safety results. Deep compliance can be translated to desired safety outcomes considering the levels of safety knowledge and expertise of workers. It can be effective in reducing occupational risks when workers are skilled enough in carrying out the procedures properly (21). In the current study, more than 99% of study workers had more than 5 years of work experience and more than 95% of them had >11 years of work experience. The difference between the results for surface compliance may be due to the mentioned issue. Moreover, empirical evidence has shown that safety compliance had a significant negative relationship with occupational injuries (13).

Both deep compliance and surface compliance show the attempts of employees to meet the organizational safety goals such as compliance with safety rules and procedures but deep compliance and surface compliance differ regarding underlying intentions and strategies and deep compliance is correlated with better safety outcomes (21).

## 5. Practical implications

The current study has important implications for employees, safety professionals, and managers in high-risk organizations. Workers should comply with safety procedures in high-levels of cognitive activities that are required to reach desired safety outcomes (21). Supervisors and safety professionals in high-risk industries should differentiate deep and surface compliance and their different contributions to safety.

Leaders’ behaviors, safety climate levels in organizations, and psychological contract of safety might affect workers’ safety compliance. Although the importance of safety leadership on safety compliance behavior has been well established, in the present study, the understanding about the effect of safety-related factors and risk perception on employees’ deep compliance and surface compliance was extended.

Organizations should motivate their workers to engage in deep compliance to carry out the safety procedures appropriately. In addition, given that surface compliance is often a prerequisite for deep compliance, it should also need to be taken into consideration. In terms of PCS, the results highlight the effects of mutual safety obligations between supervisors and employees on employees’ deep compliance. PCS also has a significant impact on safety outcomes. Risk perception has a hindrance feature for workers (29) and negatively affect both deep compliance and surface compliance. Managers and safety professionals in high-risk industries should consider appropriate measures in terms of reducing the levels of risk in these industries.

## 6. Conclusion and future research

The results of the current study support positive roles of safety-related factors such as safety leadership, safety climate, and PCS in predicting deep compliance with safety procedures. These findings can help organizations to manage factors influencing employees’ deep compliance with safety procedures. Also, it is found that risk perception can negatively affect both deep compliance and surface compliance. Both deep compliance and surface compliance were negatively associated with undesired safety outcomes.

A number of limitations need to be noted regarding this study. First, a full range of factors influencing deep compliance and surface compliance in terms of complying with safety procedures was not regarded in the current study. Second, the data were based on self-reports of employees. Future studies can investigate the effects of other factors such as workers’ intentions and motivations and the specific dimensions of safety related factors such as safety leadership and safety climate and can also use objective assessments for safety outcomes (21).

## Data availability statement

The data that support the findings of this study are available on request from the corresponding author.

## Ethics statement

The studies involving humans were approved by the Tabriz University of Medical Sciences. The studies were conducted in accordance with the local legislation and institutional requirements. The participants provided their written informed consent to participate in this study.

## Author contributions

LO and GM: conception and drafting the work. HK: acquisition. CP: drafting the work. SM: statistical analysis. All authors contributed to manuscript revision, read, and approved the submitted version.
